# Mixed release of two parasitoids and a polyphagous ladybird as a potential strategy to control the tobacco whitefly *Bemisia tabaci*

**DOI:** 10.1038/srep28245

**Published:** 2016-06-17

**Authors:** Xiaoling Tan, Nana Hu, Fan Zhang, Ricardo Ramirez-Romero, Nicolas Desneux, Su Wang, Feng Ge

**Affiliations:** 1 State Key Laboratory of Integrated management of Pest Insects and Rodents, Institute of zoology, Chinese Academy of Sciences, Beijing 100101, China; 2 Institute of Plant & Environment Protection, Beijing Academy of Agriculture and Forestry Sciences, Beijing, 100097, China; 3 Department of Plant Protection, Guizhou University, Guiyang, 550025, China; 4 Departamento de Producción Agrícola, CUCBA, Universidad de Guadalajara, Zapopan, 45100, Jalisco, México; 5 French National Institute for Agricultural Research (INRA), Univ. Nice Sophia Antipolis, CNRS, UMR 1355-7254, Institut Sophia Agrobiotech, 06903 Sophia Antipolis, France

## Abstract

A mixed species release of parasitoids is used to suppress outbreaks of tobacco whitefly, *Bemisia tabaci* (Hemiptera: Aleyrodidae); however, this biocontrol may be inhibited by interspecific interactions. We investigated the effects of mixed releases of natural enemies of *B. tabaci* on predation rates, parasite performance and adult parasitoid emergence under greenhouse conditions. We tested the polyphagous predatory ladybird *Harmonia axyridis* (Coleoptera: Coccinellidae) and two whitefly-specific parasitoids, namely *Encarsia formosa* and *Encarsia sophia* (both, Hymenoptera: Aphelinidae). *Harmonia axyridis* exhibited the lowest rates of predation when released with each parasitoid than with both parasitoid species together and showed a significant preference for non-parasitized nymphs as prey. Both *E. formosa* and *E. sophia* parasitized more *B. tabaci* when released with the ladybird than when the wasps were released either alone or mixed with the other parasitoid. We also found that the presence of *H. axyridis* significantly reduced adult parasitoid emergence; the highest rate of adult emergence was obtained with parasitoids released alone. Our results indicate that different combinations of natural enemies can influence observed rates of predation, parasitism, and parasitoid emergence. Therefore, the combination of natural enemies to be used for a particular biological control program should depend on the specific objectives.

The introduction of predatory or parasitic insects via a ‘one biological control agent–one pest’ approach has produced effective suppression of exotic pests in several applications of classical or augmentative biological control^1^,^2^. However, an increasing number of laboratory and field studies have highlighted potential problems of this simple approach, such as impacts on non-target species and, in some cases, the limited effectiveness of imported biological control agents^3^,^4^,^5^. The mixed release of multiple natural enemies as a solution to these problems has been widely explored, because mixed releases may enhance pest control in some agroecosystems, including field crops, greenhouse vegetables and organic orchards^6^,^7^,^8^,^9^. One positive feature claimed for mixed releases is that employing multiple natural enemies with different feeding patterns could ensure continued suppression of a target pest throughout its lifecycle. However, natural enemy diversity could also result in limited or reduced pest control as a result of interactions including interspecific competition, guild predation, or super parasitism among multiple biological control agents^10^,^11^,^12^,^13^,^14^,^15^,^16^.

For the tobacco whitefly, *Bemisia tabaci* Gennadius (Hemiptera: Aleyrodidae), particular attention has been paid to the use of parasitic or predatory natural enemies as biological controls^17^,^18^,^19^. This invasive whitefly, a species complex with over 30 species^20^,^21^, causes substantial damage to various crops and vegetables by direct feeding and by transmission of plant pathogens^19^,^22^,^23^,^24^,^25^. A series of outbreaks of *B. tabaci* in the Yangtze River basin and southern regions of China during the 1990s resulted in severe losses^23^,^26^,^27^,^28^,^29^. Due to conspicuous insecticide resistance^30^, many predatory or parasitic natural enemies have been introduced to suppress outbreaks of *B. tabaci*^17^,^31^,^32^,^33^,^34^. In some cases, the value measured in predation or parasitism efficiency of releasing multiple natural enemies in the management of *B. tabaci* has been found to be positive^35^,^36^. However, the successful application of mixed natural enemies can be constrained by interspecific competition among multiple biological control agents^37^,^38^ and the influence of various interactions^38^. Since no general rule has been established to predict the outcome of a mixed release of natural enemies, it is important to attempt to determine what outcome will result from the combined use of specific natural enemies to control a particular pest, prior to widespread implementation. This type of information can be very useful in the development of effective biological control programs.

In this study we were interested in determining how whiteflies are controlled by two species of parasitoids and a predator in various combinations. We studied the parasitoids *Encarsia formosa* Gahan and *E. sophia* (Girault and Dodd) (both Hymenoptera: Aphelinidae). These parasitoid species have been widely released to manage the outbreaks of whiteflies and are currently used on various greenhouse vegetables in China^35^,^39^. As heteronomus autoparasitoids, both *E. formosa* and *E. sophia* show high preference for the tobacco whitefly *B. tabaci* on various host plants, including tomato, eggplant, and poinsettia^35^,^40^,^41^. Previous studies show high competition between these two parasitoids^42^ or with other *Encarsia* species that share the same whitefly hosts^43^,^44^. Although the joint release of multiple whitefly parasitoids could improve the suppression of whiteflies, this has not always been observed (e.g. Collier & Hunter^43^). Competition between *E. formosa* and *E. sophia* and territorial colonization may modify the outcome^45^,^46^; hence the importance of testing their joint action.

As a predator we selected the Asian multicolored ladybird, *Harmonia axyridis* (Coleoptera: Coccinellidae), a generalist predator extensively employed as a biological control agent whose positive and negative effects have been reported previously^47^,^48^. Previous experiments have shown that this ladybird species exhibits significantly lower selectivity for *B*. *tabaci* and a reduced reproduction rate when fed *B*. *tabaci* whiteflies (SW, unpublished data). However, its generalist character could be helpful in controlling secondary pests and its release in combination with whitefly parasitoids might enhance whitefly control (see for example Pang *et al*.^42^, Zang *et al*.^46^). Before using this strategy it is essential to assess the possible effects of joint application of all of these control agents. Under semi-field greenhouse conditions, we assessed the parasitism and emergence rates of *E. formosa* and *E. sophia* and the predation rate of the ladybird *H. axyridis* when released alone and jointly. Our results allow us to predict outcomes were a management strategy for *B*. *tabaci* employing a mixture of these natural enemies to be implemented.

## Materials and Methods

### Insects and plants

#### Solanum lycopersicum plants

We used tomato plants var. Baofen-F1 (Changfeng Seed Co. Ltd., Xianyang City, Shaanxi, China) for *B*. *tabaci* rearing and the experiments. These plants were grown in plastic trays (55.0 × 25.0 × 20.0 cm, 10 plants per pot). The seedlings were transplanted individually into plastic flowerpots (height = 20.0 cm; diameter = 13.0 cm, one plant per pot) and maintained in artificial chambers (MH 351, Sanyo ◽, Japan) in conditions of 27 ± 1 °C, 60–65% relative humidity (RH) with a photoperiod of 14:10 (Light:Darkness). Tomato plants were used when they were approximately 30.0–35.0 cm in height with 5–7 true and fully expanded leaves.

#### Bemisia tabaci

Over 3000 pairs of *B. tabaci* were collected from greenhouse eggplants at the NOYA^®^ organic vegetable production station (40°10′38.18″N and 116°59′53.80″E), in Ping’gu district, Beijing during April 2012. The *B. tabaci* samples were classified as the Q1 biotype (MED – Q1 cryptic species) by employing a random amplification of polymorphic DNA (RAPD)-PCR (Qiu *et al*. 2003). The collected whiteflies were reared on tomato plants placed in aluminum rearing cages (50.0 × 60.0 × 45.0 cm) whose sides were covered with mesh net. Each rearing cage contained 2–3 tomato plants and 500–700 whitefly individuals. Whitefly rearing was performed at the Institute of Plant & Environment Protection, Beijing Academy and Forestry Sciences. Rearing conditions were 27 ± 1 °C, 60–65% relative humidity (RH), and a photoperiod of 14:10 (Light: Darkness). The conditions were maintained using an automatic environment management system (Suntech®, L105, Beijing, China).

#### Parasitoid wasps

Individuals of *E. formosa* (over 350 parthenogenetic female adults) and *E. sophia* (317 females and 103 males) were collected from a tobacco field at the Wang’jia’yuan Biodiversity Research Station (40°10′45.30″N and 116°2′38.27″E), Chang’ping district, Beijing during April 2012. *E. sophia is* known as a complex including multiple cryptic species^49^. We classified *E. sophia* as the Pakistan and Spanish genotype by using the mitochondrial cytochrome oxidase subunit I gene^50^.The wasp populations were maintained under the same laboratory conditions as the whiteflies. Each rearing cage contained 4–6 tomato plants infested with whitefly nymphs, and was maintained with 200–220 females *and* 30–50 males of *E. sophia* or 150–200 *E. formosa* individuals, each species kept separately. Tomato plants harboring whitefly nymphs were replaced every three days to enable reproduction of the wasps. Both wasp species were employed in the experiments after living for three generations under our laboratory conditions in order to standardize host preference. Five day old wasps were used in experiments to ensure they had reached sexual maturity^41^,^51^. For pre-mating, a pair of 5 day old *E. sophia* adults were introduced into a plastic petri dish (D = 4.5 cm). After 24 h, the mated female adults were collected for following tests. We used unmated (parthenogenetic) *E. formosa* females and pre-mated *E*. *sophia* females. Prior to the experiments, the wasps were provided with honey droplets as a food supplement.

#### Harmonia axyridis

A total of 110 pairs of *H. axyridis* adults were captured using a rape-pollen trapping chamber in Beijing Botanic Garden (39°59′29.65″N and 116°12′34.33″E), Haidian district, Beijing during May 2012. The ladybirds were transported and maintained under the same environmental conditions as the wasps in a rearing chamber at the Wang’jia’yuan Biodiversity Research Station. The ladybirds were reared in custom-made cages (50.0 × 50.0 × 60.0 cm, employing a 60-mesh fabric net and aluminum frames, with a ladybird density of 50–70 pairs of adults or 100–140 larvae per cage) and provided with an abundant daily supply of nymphs of the aphid *Megoura japonica* (Hemiptera: Aphididae) Matsumura on house bean *Vicia faba* var. Liying (Xinfeng seed Co. ltd, Beijing) sprouts. Ladybirds were raised for 3 generations under laboratory conditions to standardize food preference before use in experiments. Ladybirds were used in experiments at the 4^th^ instar stage; at this stage ladybird larvae consume a substantial amount of prey^52^.

### Semi-field experiments

Our experiments were performed in a greenhouse (under natural environmental conditions) located at the NOYA^®^ organic vegetable production station (40°10′38.18″N and 116°59′53.80″E). This greenhouse was divided into 6 isolated plots (Length: 12 m, Width: 7.0 m); limited by 80-mesh fabric net, 120 evenly spaced tomato plants were transplanted into each plot (12 plant per row × 10 rows). The greenhouse experiments were replicated three times during the course of the study: 9–27 July; 1–17 August; and 21 August–8 September.

Based on pilot study observations, six natural enemy combination treatments were established at each plot: (i) 30 *E. formosa* females (thereafter F), (ii) 30 *E. sophia* females (thereafter S), (iii) 30 *E. formosa* females and 10 *H. axyridis* (thereafter F + H), (iv) 30 *E. sophia* females and 10 *H. axyridis* (thereafter S + H), (v) 15 *E. formosa* females and 15 *E. sophia* females (thereafter F + S)] and, (vi) 15 *E. formosa* females, 15 *E. sophia* females and 10 *H. axyridis* (thereafter F + S + H).

The observations were conducted in different greenhouse plots, each employing one natural enemy combination. In each plot, 30 tomato plants with 9–10 true and fully expanded leaves were randomly selected. Each selected plant was covered with a cylindrical net cage (height = 50.0 cm; diameter = 30.0 cm, composed of 80-mesh fabric net and aluminum frames). Then, 250 pairs of *B. tabaci* adults were released onto each caged tomato plant and allowed to oviposit. After 48 h, the *B. tabaci* adults were removed from the cages. Then, after 14 days, all *B. tabaci* except third-instar nymphs (identified as light green or yellow elliptic body covered with wax, and body size of approximately 0.5 mm), the most suitable instar for parasitism, were removed from the plants by using a dissecting needle and smooth brush. Natural enemies were then introduced into the cages. At this point, six leaves of each plant were randomly selected and marked with cardboard labels (three with black labels and three with red labels). The number of nymphs on the six leaves was recorded at the time of labeling (i.e. the initial number of nymphs).

Forty-eight hours after the introduction of natural enemies, the three leaves with black labels from each tomato plant were removed and transferred to the laboratory. All nymphs on the leaves were assessed to determine if they had been preyed upon or not. To assess predation events, we looked for the remains of the body of the nymphs using a stereoscope (SteREO Discovery V20, Zeiss, Germany). We recorded the number of preyed upon nymphs and calculated percentages of predation (number of preyed upon nymphs/number of initial nymphs). All individual nymphs and nymph remains on the leaves were kept separate to assess parasitism using molecular analyses^53^. This method is based on the cytochrome oxidase subunit I (COI) gene of mitochondrial DNA (mtDNA) and enables the detection and identification of *E. formosa* and *E. sophia* in *B. tabaci* nymphs at very early developmental stages^53^. By this method the total numbers of non-parasitized and parasitized whitefly nymphs (by *E. sophia*, *E. formosa*, or both) on the black labeled leaves were determined and the percentage (=number of parasitized nymphs/initial number of nymphs) of *B. tabaci nymphs* parasitized by *E. sophia*, *E. formosa*, or both was calculated.

To determine the rates of emergence of *E. sophia* and *E. formosa* in the various treatments, the three leaves marked with red labels were isolated in 600-ml plastic bags with over 400 holes drilled for ventilation after some parasitized whitefly nymphs had turned black, indicating that the parasitoids had developed to the pupal stage. The number of black nymphs was counted and recorded as the total number of parasitized nymphs. The marked leaves were then checked daily and the number of newly emerged *E. sophia* and *E. formosa* were recorded. The number of emerged parasitoids (*E. sophia*, *E. formosa*, or both) from all three leaves was recorded and used to calculate the percentage of adult emergence (=total number of emerged adults/total number of parasitized nymphs).

### Statistical analysis

The percentages of total *B. tabaci* nymphs preyed upon by *H. axyridis* and percentages of adult parasitoid emergence were compared using multiple factorials ANOVA across multiple natural enemy combinations as independent factors and 3 different temporal replications as partial factors. Multiple comparisons were performed via a Tukey HSD test (*P* = 0.05). The predation rate on non-parasitized whitefly nymphs was compared against the predation rate on parasitized nymphs using a Chi-square test (*P* = 0.05). Arcsine square root transformation was applied to the percentage data before statistical analysis. ANOVA and Tukey analyses were processed using the statistical analysis software SPSS 18.0^54^.

## Results

### Predation of Harmonia axyridis

**Figure 1:**
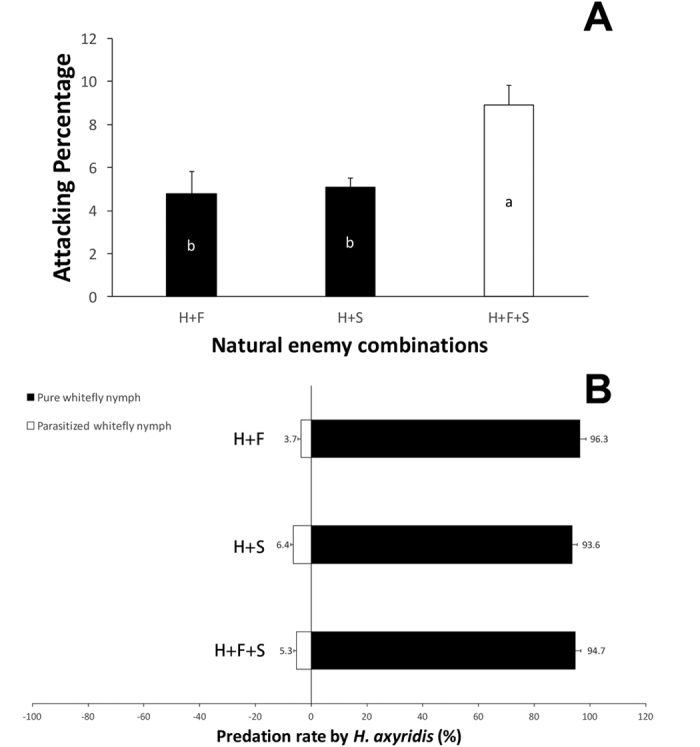
The Attacking percentage (**A**) and predation rate by *Harmonia axyridis* which released with *Encarsia formosa*, *E. sophia* or both of them respectively (**B**). The error bars showed in the charts are standard error. The different letters with the columns in chart A indicate the significant differences among different natural enemy combinations in P = 0.05 by Tukey HSD test.

The rates of predation of *H. axyridis* on whitefly nymphs were significantly different depending upon the natural enemy combination present (Fig. 1). Significantly more whitefly nymphs were preyed upon by the ladybird when released with both parasitoids simultaneously than when released with either *E. formosa* or *E. sophia* individually (Fig. 1A, *F* = 12.36, *P* < 0.01). The temporal replications did not show any significant influences on predation (*F* = 0.76, *P* = 0.55). *Harmonia axyridis* also showed a significant preference for non-parasitized whitefly nymphs in all treatments where parasitoids were present (Fig. 1B, H + F: *X*^2^ = 81.03; H + S: *X*^2^ = 84.61; H + F + S: *X*^2^ = 86.64; all P < 0.01). No significant difference was found between the predation rates on non parasitized nymphs across treatments (Fig. 1B) (*F* = 1.113, *P* = 0.247), and no significant differences were found between temporal replications (*F* = 0.86, *P* = 0.36).

### Parasitic proportion of parasitoids

**Figure 2:**
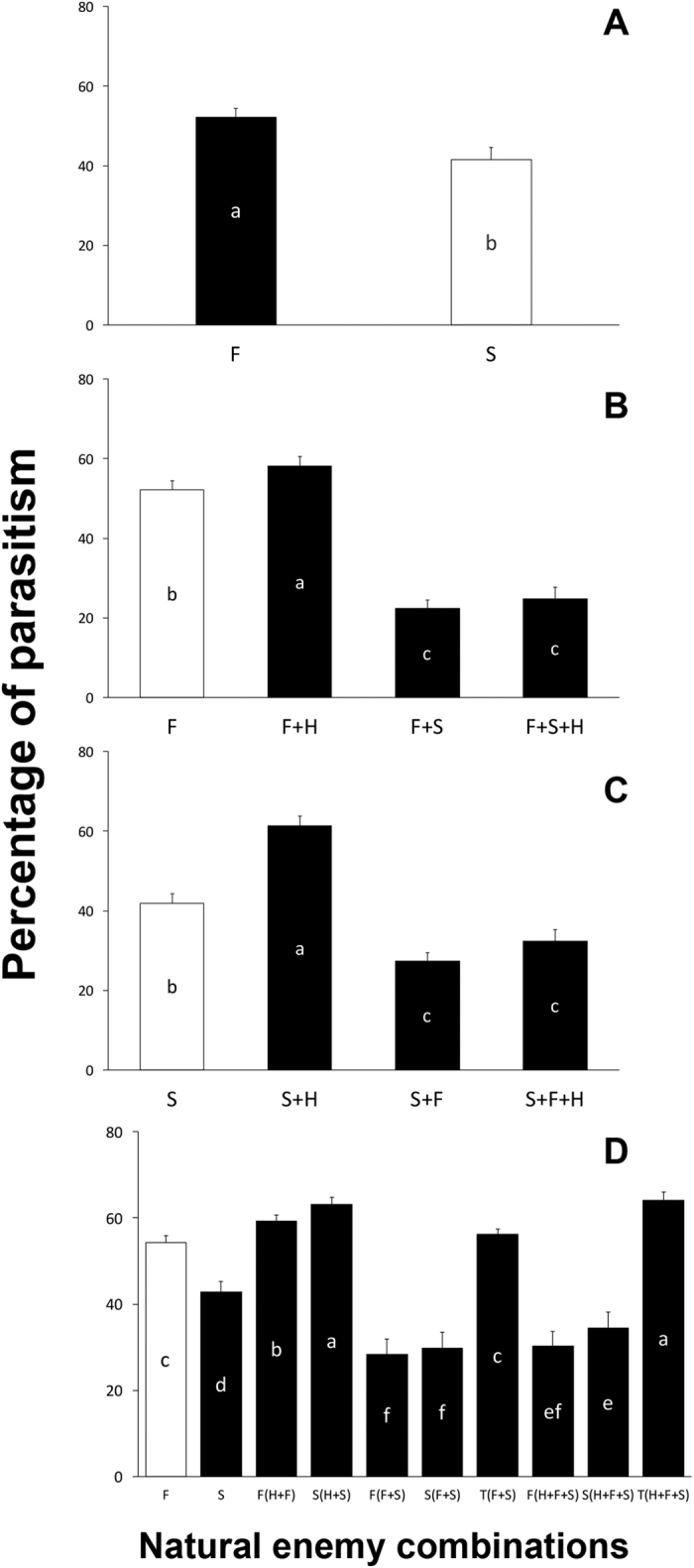
The percentage of parasitism of two parasitoids in different natural enemy combination treatments. (**A**) The percentage of parasitism of *Encarsia formosa* and *E. sophia* when t released independently; (**B**) the percentage of parasitism of *E. formosa* when released with different natural enemies; (**C**) the percentage of parasitism of *E. sophia* when released with different natural enemies; (**D**) the the percentage of parasitism of two parasitoids when released with different natural enemies. The abbreviated letters in the figure means as: F = *E. formosa*; S = *E. sophia*; H = *Harmonia axyridis*; T = total parasitic percentage. The error bars on the top of the columns are standard errors. The different letters within the columns indicate the significant differences among different natural enemy combinations in P = 0.05 by Tukey HSD test.

When each was released alone, *E. formosa* parasitized significantly more *B. tabaci* than *E. sophia* did (Fig. 2A) (*t* = 101.47, *P* < 0.01), a difference invariant over temporal replications (*F* = 1.31, *P* = 1.23). The percentage of parasitism by *E. formosa* was significantly higher when this wasp was released alone relative to treatments where it was released with *E. sophia* and with both *E. sophia* and *H. axyridis* (Fig. 2B) (*F* = 76.87, *d.f*. = *P* < 0.01) and did not vary across temporal replications (*F* = 0.98, *P* = 0.44). However, the parasitism rate of *E. formosa* was significantly higher when released in combination with *H. axyridis* than when alone(Fig. 2B). Similarly, the percentage of parasitism by *E. sophia* alone was significantly higher than parasitism observed when *E. sophia* was released along with *E. formosa* or along with *E. formosa* and *H. axyridis* together (Fig. 2C). The parasitism rate shown by *E. sophia* when released along with *H. axyridis* was also significantly higher than *E. sophia* released alone. (Fig. 2C) (*F* = 85.13, *P* < 0.01) this difference was also unchanged over temporal replications (*F* = 1.17, *P* = 0.28). An overall view of the parasitism outcome across different combinations is provided by Fig. 2D. We can identify three main outcomes depending upon the natural enemy combination. First, the highest rate of parasitism was exhibited by each parasitoid released with only the predator (Fig. 2D). In a second group we found lower rates of parasitism for each wasp species acting alone (Fig. 2D). A third group showing the lowest rates of parasitism ocurred for those treatments where both parasitoids were present, with or without the predator (Fig. 2D).

### Adult parasitoid emergence

**Figure 3:**
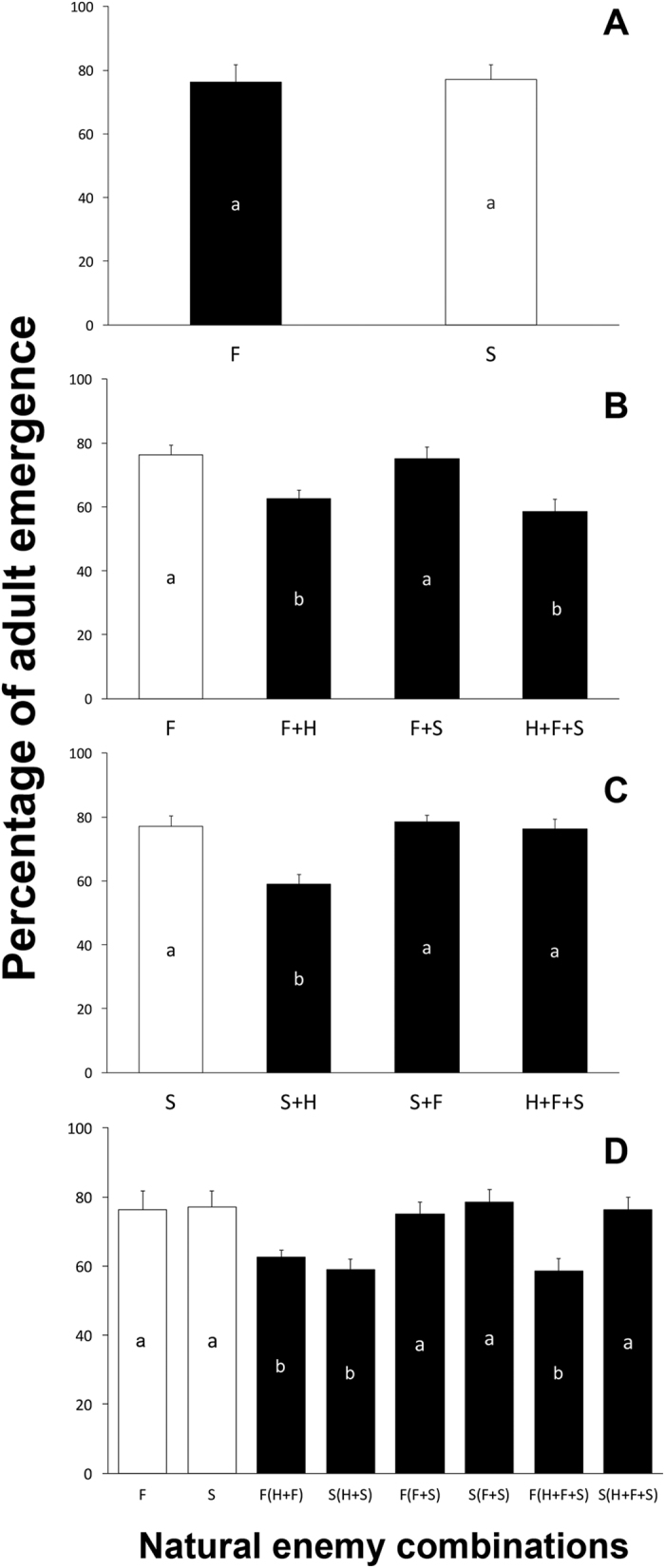
The percentage of adults emergence of two parasitoids in different natural enemy combination treatments. (**A**) the percentage of adults emergence of *Encarsia formosa* and *E. sophia* when released independently; (**B**) the percentage of adults emergence of *E. formosa* when released with different natural enemies; (**C**) the percentage of adults emergence of *E. sophia* when released with different natural enemies; (**D**) the the percentage of adults emergence of two parasitoids when released with different natural enemies. The abbreviated letters in the figure means as: F = *E. formosa*; S = *E. sophia*; H = *Harmonia axyridis*. The error bars on the top of the columns are standard errors. The different letters within the columns indicate the significant differences among different natural enemy combinations in P = 0.05 by Tukey HSD test.

Both parasitoid species showed similar percentages of parasitoid emergence (Fig. 3A) (*t* = 0.157, *P* = 0.0876) this was not influenced by temporal replications (*F* = 1.04, *P* = 0.19). *E. formosa* released alone exhibited an emergence rate similar to that which occurred when released along with *E. sophia* (Fig. 3B). However, the emergence rate of *E. formosa* alone was significantly higher that the rates exhibited by this species when released with *H. axyridis* or with both *H. axyridis* and *E. sophia* (Fig. 3B) (*F* = 93.13, *P* < 0.01) and not influenced by temporal replications (*F* = 1.26, *P* = 1.14). *E. sophia* rates of adult emergence when released alone were similar to those displayed when released along with *E. formosa* or with both *E. formosa* and *H. axyridis* (Fig. 3C). However, significantly more *E. sophia* adults emerged when *E. sophia* was released alone than when released with the predator (Fig. 3C) (*F* = 64.13, *P* < 0.01)this difference was also not influenced by temporal replication variation (*F* = 1.06, *P* = 0.19). The percentages of adult emergence divide into two principal groups. The group with the highest rates of adult emergence includes those treatments where the wasps were released alone or in combination with the other parasitoid wasp (Fig. 3D). The lowest levels of adult emergence were obtained when each wasp was released in combination with the predator, or when both wasps were released with the predator (Fig. 3D) (*F* = 101.47, *P* < 0.01). This was not influenced by the temporal replications (*F* = 1.01, *P* = 0.17).

## Discussion

Our results showed that the release of the predator along with both parasitoid species resulted in a higher rate of whitefly predation relative to the other treatments. Thus, more whitefly nymphs will be preyed upon when the predator is released with both parasitoids, a positive outcome for pest control. These results indicate that the presence of both parasitoids induces a higher rate of predation. A possible explanation for the increased rate of predation is some cue provided by parasitoids, a cue which might be physical (e.g. motion detection of the wasps) or biochemical (e.g. semiochemical traces related to the wounds produced by parasitoids when host-feeding). We know that some predators are able to detect biochemical information or fingerprints of conspecifics^55^,^56^. The role of physical characteristics of prey (e.g. color and size) in detection and preference by a predator has also been discussed^14^,^57^. The finding of more prey consumed contrasts with previous findings that increasing the number of biological control agents does not necessarily produce positive effects on pest suppression^58^. It seems important to evaluate specific combinations of natural enemies before use on biological control programs due to the observed heterogeneity of effects on pest suppression^59^.

We found that the predator significantly preferred non-parasitized nymphs. Our study used third-instar nymphs exposed at the same time to parasitoids and predators, requiring predators to choose between preys very similar in appearance. Multiple factors have been proposed to explain a predator’s preference for parasitized over non-parasitized nymphs. Examples are size and color of prey^57^ and mechanical and physiological changes related to parasitism^14^. Our results indicate that factors related to physiological changes following parasitism may play an important role in the predator’s preference. Other predators have been reported to show similar a preference for non-parasitized over parasitized hosts (e.g. Colfer and Rosenheim^12^; Velasco-Hernandez *et al*.^14^).

Our results show that when released alone, *E. formosa* exhibited higher rates of parasitism than *E. sophia* (Fig. 2A). These results are in agreement with those reported by Pang *et al*.^42^ under laboratory conditions. Both experiments indicate that the wasp *E. formosa* will be able to parasitize more nymphs than *E. sophia* when each is released alone. However, the rate of parasitism of each parasitoid when alone was surpassed by the rates obtained when each parasitoid was released along with the predator (Fig. 2B,C). Thus, the parasitoids increased their rates of parasitism in the presence of the predator. Similar results have been reported for other parasitoid species in the presence of other predators. For example, the parasitoid *Aphidius ervi* (Braconidae, Aphidiinae) attacking the pea aphid *Acyrthosiphon pisum* (aphididae, Macrosiphoninae) in the presence of the predator *Coccinella septempunctata*, exhibited a higher rate of ovipositions^60^. In a similar effect, the parasitoid *Eretmocerus eremicus* (Hymenoptera: Aphelinidae) increases its number of ovipositions in the presence of the predator *Geocoris punctipes* (Hemiptera: Lygaeidae)^15^. The later authors suggest that this increase in ovipositions may be a response to wasp predation (i.e. intraguild predation or IGP). Although in the current study IGP was low (Fig. 1B), the parasitoids could be increasing their rate of parasitism to counteract the effect of competition for the shared whitefly nymph prey. This type of response to competitive situations has been previously documented; the introduction of a stronger competitor influencing a weaker competitor to become more efficient (Griffen & Williamson^61^; Mullan *et al*.^62^, but see Chailleux *et al*.^9^).

We note that the lowest rates of parasitism were found when parasitoids were released together, regardless of the presence of the predator. The joint presence of the two parasitoid species inhibits their performance. It is possible that competition between these species as previously documented^42^,^46^ explains this reduction in performance. Conditions such as heterospecific host-feeding^42^ or the sex-ratio of *E. sophia*^46^ could play an important role in the parasitism performance of these species when released together.

Contrasting results were found when we analyzed the rates of adult parasitoid emergence (Fig. 3). Higher rates of adult emergence were obtained when parasitoids were released without predators, whether alone or mixed (Fig. 3B,C). Predator presence has a negative effect on the adult emergence of both parasitoid species. In our experimental design the adult emergence rate was calculated by dividing the number of emerged wasps by the number of nymphs turned dark by the presence of the parasitoid. Thus, the measured reduction in the rate of adult emergence is due to mortality during the immature stage and not to predation. However, the predator had access to the parasitized nymphs for 48 hours and could have hurt the immature wasps, increasing mortality prior to the adult stage^14^,^63^. This is the best explanation we have now, as predator absence is the only distinctive factor in treatments displaying higher adult wasp emergence. Of course, this hypothesis remains to be tested.

It appears that the effectiveness of mixed releasing of these biological control agents is not influenced by the combination of agents, but by the ratio between predators and parasitoids. Regulation of the complex structure, especially the population scales of primary species, may direct influence the changing of the food web around them^64^,^65^. An imbalanced ratio between intra guild predators and parasitoids may increase the risks of interspecific cannibalism and decrease the efficiency of pest management^66^. The best way to avoid this problem is to identify the most effective ratio of these biological control agents. Here, we did not attempt to optimize the ratio of *H. axyridis* to *E. formosa* and *E. sophia* for the best *B. tabaci* suppression. A future experiment will be designed to explore the optimal predators/parasitoid ratio for mixed natural enemy releases. Our results seem help useful for the design of biological pest control plans because they can assist in exploring scenarios that employ different natural enemy combinations. For example, if the aim is simply to reduce the population of whiteflies, it is possible that the combination of one wasp and the predator is the best choice. This is due to the high rate of parasitism obtained with this combination, and the predator’s high preference for non-parasitized nymphs. However, if establishment of either of the parasitoids is most important, our results indicate that the best option would be to release the parasitoids individually or together, absent the predator. This is because predator presence reduces the rate of emergence of parasitoids. Of course, we only assessed the interaction between parasitoids and predatory ladybirds in terms of a single generation of parasitoid progeny and predation measured during a very limited time span. The actual consequences of mixed releasing of natural enemies according to our suggestion may prove different in the field where consecutive and overlapping generations of natural enemies occur. The combination of natural enemies studied here needs to be tested in additional experiments in more natural conditions, taking into account the effects of multiple overlapping generations of natural enemies.

## Additional Information

**How to cite this article**: Tan, X. *et al*. Mixed release of two parasitoids and a polyphagous ladybird as a potential strategy to control the tobacco whitefly *Bemisia tabaci*. *Sci. Rep*. **6**, 28245; doi: 10.1038/srep28245 (2016).
